# Apolipoprotein E Gene Variants and Risk of Coronary Heart Disease: A Meta-Analysis

**DOI:** 10.1155/2016/3912175

**Published:** 2016-10-27

**Authors:** Min Xu, Jun Zhao, Yu Zhang, Xu Ma, Qiaoyun Dai, Hong Zhi, Bei Wang, Lina Wang

**Affiliations:** ^1^Key Laboratory of Environmental Medicine Engineering, Ministry of Education, Department of Epidemiology and Biostatistics, School of Public Health, Southeast University, Nanjing, China; ^2^Graduate School of Peking Union Medical College, Beijing, China; ^3^National Research Institute for Family Planning, Beijing, China; ^4^Centers for Disease Control and Prevention, Zhejiang, China; ^5^Department of Cardiology, ZhongDa Hospital, Southeast University, Nanjing, Jiangsu, China

## Abstract

*Objectives*.* Apo E* genes involved in lipoprotein synthesis and metabolism are considered one of the candidates to CHD. However, the results remain conflicting.* Methods*. We performed this meta-analysis based on 30 published studies including 11,804 CHD patients and 17,713 controls.* Results*. Compared with the wild genotype E3/3, the variant genotypes* ApoE*E3/4 and E4/4 were associated with 22% and 45% increased risk of CHD, respectively (E3/4 versus E3/3: OR = 1.22, 95% CI = 1.15–1.29; E4/4 versus E3/3: OR = 1.45, 95% CI = 1.23–1.71). Besides, compared with *ε*3 allele, carriers with the *ε*4 allele had a 46% increased risk of CHD (OR = 1.46, 95% CI = 1.28–1.66), while the *ε*2 had no significantly decreased risk of CHD. In the subgroup analysis by ethnicity, *ε*4 had a 25% increased risk of CHD in Caucasians (OR = 1.25, 95% CI = 1.11–1.41), and the effects were more evident in Mongolians (OR = 2.29, 95% CI = 1.89–2.77). The *ε*2 allele had a decreased risk of CHD in Caucasians (OR = 0.84, 95% CI = 0.74–0.96), but not in Mongolians.* Conclusions*. The analysis suggested that* ApoEε*4 mutation was associated with the increased risk of CHD, while* ApoEε*2 allele had a decreased risk of CHD just in Caucasians.

## 1. Introduction

Coronary heart disease (CHD) as a multifactorial disease caused by genetic and environmental factors became one of the leading causes of mortality and morbidity worldwide, especially in the developed countries [1]. Previous studies provided evidence that risk factors for CHD, including diabetes mellitus, smoking, and arterial hypertension, would contribute to rapid process of clinical events such as myocardial infarction (MI), ischemic heart failure, and death [2, 3]. Moreover, apart from the above risk factors, population-based studies have reported that genetic susceptibility accounts for around 50% of the risk for CHD, which suggested that the host genetic background plays an important role in the onset and development of CHD as well [4, 5]. The determination of blood lipid and lipoprotein levels is one of the coronary risk factors. Apolipoprotein genes involved in lipoprotein synthesis and metabolism play imperative roles in studying the susceptibility to CHD and cerebrovascular disease [6–11].

In 1992, Dallongeville et al. tested the consistent relationship between plasma TG levels and* Apo E* phenotype among 45 different populations in a meta-analysis [12]. Another meta-analysis including 14 studies showed that subjects with *ε*4 allele were associated with 26% increased risk of CHD compared with *ε*3 allele [13]. Since the publication of these two meta-analyses, numerous studies have appeared in recent years [14–17]. However, differences in study design, end point validation, choice of subjects, and limited statistical power have led to different results of* Apo E* genotypes on CHD risk in the general population. Therefore, the present meta-analysis is designed to derive a more plausible estimation.

## 2. Methods

### 2.1. Studies Selection

We search the electronic databases PubMed, Web of Science, Embase, Wanfang, China National Knowledge Internet (CNKI), and VIP for relevant English language articles from Jan 1, 2000, to Mar 1, 2016, using the following index terms:* Apo E* and polymorphism(s) single nucleotide polymorphism (SNP), variant, mutation and coronary artery disease, coronary heart disease, CAD, and CHD. Hand searches were also performed.

### 2.2. Inclusion Criteria

The inclusion criteria were as follows: (1)* Apo E* gene polymorphism in CAD or CHD; (2) case-control studies; (3) studies with full-text articles; (4) sufficient data for calculating an odds ratio (OR) with 95% confidence interval (CI); (5) not republished data.

### 2.3. Data Extraction

All the data were carefully extracted from all eligible publications independently by two authors. The following information was collected: the first author's name, date of publication, country, study design, major CHD end point, selection of the controls, genotyping methods, allele frequencies of *ε*2, *ε*3, and *ε*4, and genotype distribution in case-patients and controls. Definition of CHD end points includes MI, angina pectoris, percutaneous transluminal coronary angioplasty, coronary artery bypass graft surgery, and severe stenosis on coronary angiography. In most studies that included case-patients with coronary artery disease, diagnosis was based on angiographically documented luminal stenosis (≥50%) in at least 1 of the 3 major coronary arteries.

### 2.4. Quality Score Assessment

To determine the methodological quality of each study, we used the Newcastle-Ottawa scale (NOS). The NOS ranges between zero (worst) up to nine stars (best). Two authors of this article independently assessed the qualities of included studies. Disagreement was resolved by discussion.

### 2.5. Meta-Analysis

The risks (odds ratios, ORs) of CHD associated with the* Apo E* polymorphisms were estimated for each study with the software Stata12.0. The risk of the variant genotypes E2/2, E2/3, E2/4, E4/3, and E4/4 was estimated compared with the genotype E3/3 homozygotes. In addition to comparisons for total subjects, studies were categorized into different subgroup analyses according to the ethnicity. We estimated the between-study heterogeneity across the eligible comparisons using the Cochrane* Q*-test and the heterogeneity was considered significant for *P* < 0.1. Fixed effect or random effect was used to calculate pooled effect estimates. Random effects incorporate an estimate of the between-study variance and tend to provide wider confidence intervals, when the results of the constituent studies differ among themselves. In the absence of between-study heterogeneity, the two methods provide identical results.

Publication bias was evaluated by funnel plot and Begg's and Egger's tests. The Hardy-Weinberg equilibrium (HWE) was tested by a goodness-of-fit *χ* ^2^ test to compare the observed genotypes frequencies with the expected ones among control subjects. For sensitivity analyses, we examined whether the excluding studies with substantial deviation from HWE among controls affected our pooled estimates of ORs.

Finally, we use the following formula to estimate the fail-safe number:

(1) Nfs0.05=∑Z1.64×2−K,Nfs0.01=∑Z2.33×2−K.

*K*  is the number of included studies and *Z* is the *Z* value of the independent study. The result is obtained from the software SAS 9.2.

## 3. Result

### 3.1. Studies Characteristics

**Figure 1:**
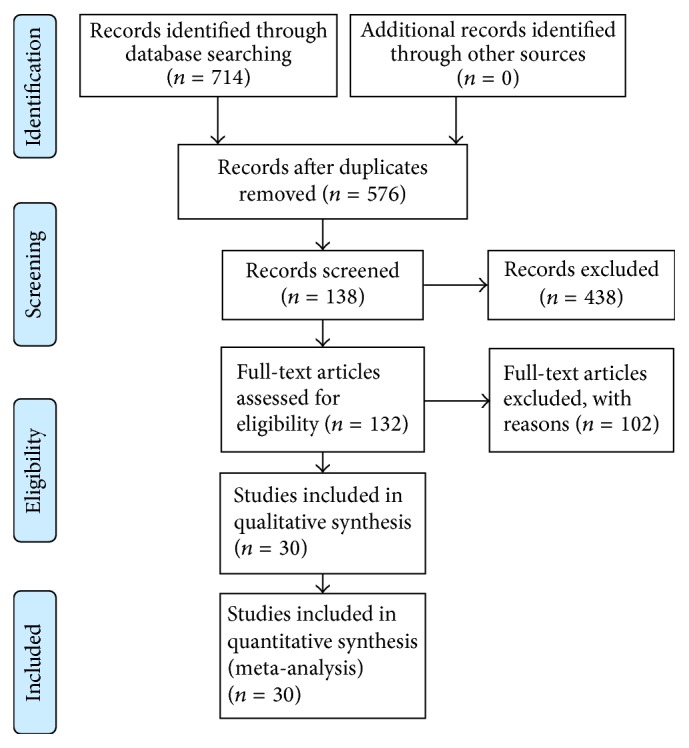
The flow diagram of study selection.

714 studies were searched, among which 30 studies were included in the final meta-analysis [18–47]. The study selection process is detailed in Figure 1.

Given in Table 1 were the lists of number of cases, controls, HWE, and the NOS score of these 30 case-control studies. All studies indicated that the distribution of genotypes in the controls was consistent with HWE, except for 7 studies [29, 35, 37, 39, 40, 45, 46] (*P* < 0.05). According to the quality criteria, there were 21 studies with high quality (NOS score > 6).

Tables 2 and 3 showed the frequency distributions of the* Apo E* alleles and genotypes in the cases and controls.

### 3.2. Meta-Analysis Results

**Figure 2:**
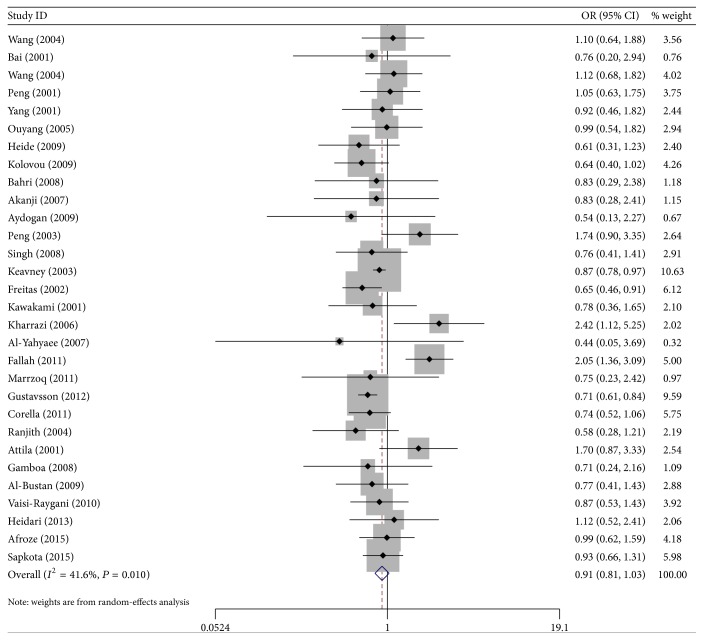
ORs of CHD associated with* Apo E* for the allele *ε*2 compared with the *ε*3.

**Figure 3:**
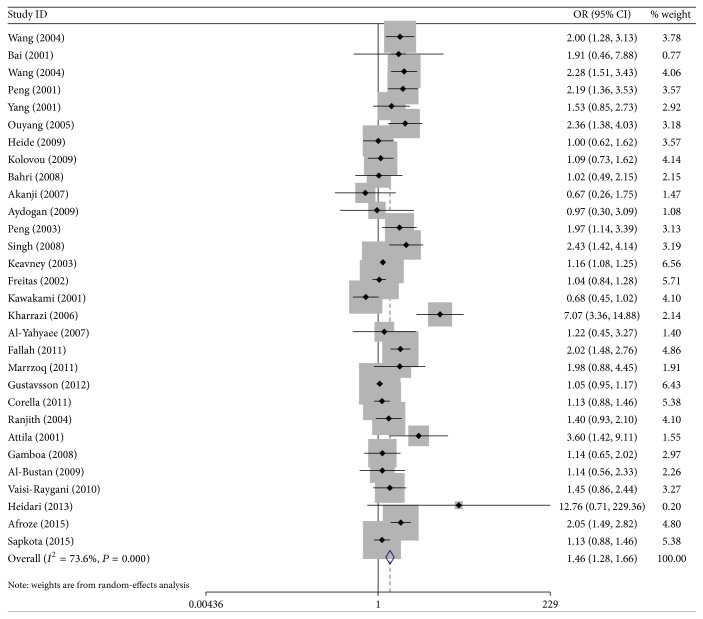
ORs of CHD associated with* Apo E* for the allele *ε*4 compared with the *ε*3.

Figures 2 and 3 showed the ORs on CHD associated with* ApoEε*2 alleles and* ApoEε*4 alleles compared with the *ε*3 alleles in individual studies.

Overall, in the dominant model, individuals carrying the* ApoE*E2/2, E2/3, and E2/4 genotypes did not have significant risk of CHD compared with individuals with the E3/3 genotype (E2/2 versus E3/3: OR = 1.03, 95% CI = 0.79–1.34; E2/3 versus E3/3: OR = 0.89, 95% CI = 0.76–1.06; E2/4 versus E3/3: OR = 1.04, 95% CI = 0.87–1.23), However, compared with E3/3 genotype, the variant genotypes* ApoE*E4/4 and E3/4 were associated with increased risk of CHD in 30 studies in the genetic models (E3/4 versus E3/3: OR = 1.48, 95% CI = 1.26–1.75; E4/4 versus E3/3: OR = 1.45, 95% CI = 1.23–1.71).

In the subgroups, the results showed evidence of significant association between* Apo E* gene polymorphism and CHD risk in the genetic model of E3/4 and E4/4 compared with the genotype E3/3 in eight Mongolian studies (E3/4 versus E3/3: OR = 1.73, 95% CI = 1.02–2.93; E4/4 versus E3/3: OR = 2.78, 95% CI = 1.35–5.72), while the variant genotypes* ApoE*E2/2, E2/3, and E2/4 were not associated with the increased risk of CHD (E2/2 versus E3/3: OR = 1.08, 95% CI = 0.39–3.00; E2/3 versus E3/3: OR = 1.16, 95% CI = 0.91–1.49; E2/4 versus E3/3: OR = 1.38, 95% CI = 0.62–3.11). In 22 Caucasians studies, significant associations were found in three genetic models (E2/3 versus E3/3: OR = 0.81, 95% CI = 0.67–0.98; E3/4 versus E3/3: OR = 1.38, 95% CI = 1.17–1.62; E4/4 versus E3/3: OR = 1.51, 95% CI = 1.12–2.04); carriers with* ApoE*E2/2 and E2/4 were not associated with the significant risk of CHD (E2/2 versus E3/3: OR = 1.03, 95% CI = 0.78–1.35; E2/4 versus E3/3: OR = 1.02, 95% CI = 0.86–1.22).

Besides, carriers with* ApoEε*2 allele had no significantly decreased risk of CHD compared with individuals with the *ε*3 allele in the random-effect model (OR = 0.91; 95% CI = 0.81–1.03). Stratified analysis on the descent also showed no evidence on the *ε*2 allele variant and CHD risk in Mongolians (OR = 1.18, 95% CI = 0.94–1.46), but there had a decrease risk in Caucasians (OR = 0.84, 95% Cl = 0.74–0.96). In addition, compared with the* ApoEε*3 allele, carriers with the *ε*4 allele had a 46% increased risk of CHD in the random-effect model (OR = 1.46, 95% CI = 1.28–1.66), and the effects were more evident in the Mongolians (the random-effects model OR = 2.29, 95% CI = 1.89–2.77) and mild in the Caucasians (the fixed-effects model OR = 1.25, 95% CI = 1.11–1.41).

### 3.3. Sensitivity Analysis

We conducted a sensitivity analysis on the* Apo E* polymorphisms and risk of CHD excluding studies deviating from HWE among controls. The pooled ORs estimates were similar with that of excluded studies, so the results were not shown.

### 3.4. Bias Diagnostics

**Figure 4:**
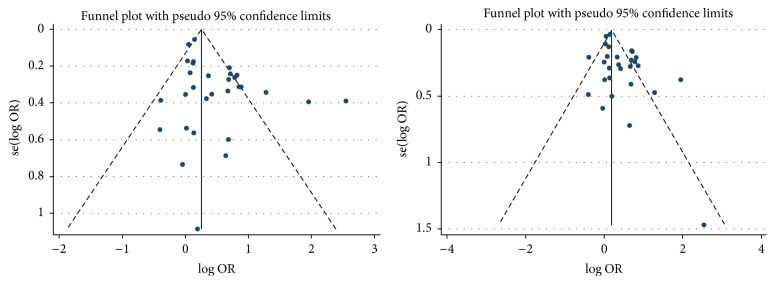
The funnel plot of the* Apo E* allele *ε*2 and *ε*4 compared with the *ε*3.

For the* ApoEε*2 versus *ε*3 allele, the shape of the funnel plot seemed symmetrical (Figure 4), and then Egger's test showed no evidence of publication bias (*P* = 0.211), and the fail-safe number also showed no publication bias in this meta-analysis (N_fs_(0.05) = 381.51, N_fs_(0.01) = 173.87).

For the* ApoEε*4 compared with *ε*3 allele, the shape of the funnel plots seemed asymmetrical (Figure 4), and Egger's test revealed there was a significant publication bias (*P* = 0.003). By using the trim and fill method, we showed that OR and 95% CI did not change. Besides, the fail-safe number also suggested that the result of the meta-analysis was stability (N_fs_(0.05) = 1218.31, N_fs_(0.01) = 588.44).

## 4. Discussion

Apolipoprotein E (*Apo E*) is one of the most major apolipoproteins in the central nervous system, with functions of neurons repair.* Apo E* genetic variants showed significant associations with the risks of nervous system degenerative diseases, including Alzheimer's disease, vascular dementia, and cerebrovascular disease [48, 49].* Apo E* gene as a receptor-binding ligand mediating the clearance of chylomicron and remnants of very-low-density lipoprotein cholesterol from plasma also plays a major role in the metabolisms of cholesterol and triglyceride. Functional variants of genes encoding lipoproteins are responsible in part for between-individual variation in the plasma levels of lipoproteins.

*Apo E* with 3 major isoforms, E2, E3, and E4, which are coded by the corresponding alleles, *ε*2, *ε*3, and *ε*4, has 6 genotypes, E2/2, E2/3, E2/4, E3/3, E3/4, and E4/4. Compared with *ε*3 carriers, carriers of the *ε*2 allele, which has defective receptor-binding ability, have lower circulating cholesterol levels and higher triglyceride levels, whereas carriers of the *ε*4 allele appear to have higher plasma levels of total and low-density lipoprotein cholesterol [50–52]. In serum or plasma cholesterol of healthy Caucasians,* Apo E* polymorphisms accounted for approximately 2%–11% of the total [53]. Recent evidences also indicated that* Apo E* may play additional roles in the development of CHD through macrophage cholesterol efflux, platelet aggregation, and allele-specific antioxidant and immune activities [53–55].

A lot of epidemiologic studies have investigated the relation between* Apo E* genotypes and CHD risk in the general population.* Apo E* polymorphisms are believed to confer susceptibility to CHD risk. In 1992, a meta-analysis of 27 studies reported that the subjects carrying the *ε*2 and *ε*4 alleles had, respectively, lower and higher plasma cholesterol values than subjects carrying the E3/3 genotype, which suggested that the* ApoEε*4 allele may, in individuals with the* ApoE*E4/3 phenotype, be a risk factor of cardiovascular disease [12]. The last meta-analysis of 14 published case-control studies in 2015 showed that carriers with* POEε*2 allele were associated with the decreased risk for CHD (OR = 0.82, 95% CI: 0.75–0.90) compared with those carrying *ε*3 allele, while those with *ε*4 allele had a significant increased risk for CHD (OR = 1.34, 95% CI: 1.15–1.57) [15].

In this 30 studies' meta-analysis including 11,804 CHD patients and 17,713 controls, we identified a significant increased risk for CHD among carriers of the* ApoE*E3/4 and E4/4 genotypes compared with carriers of the E3/3 genotype, but no significant evidence was found between the variant genotypes of* ApoE*E2/3, E2/4, and E2/2 and CHD risk. In the stratified analyses by descent, for the *ε*4 allele genetic variant, the effect was more evident in the Mongolians group and mild in Caucasians group, which showed that the* ApoEε*4 allele genetic variant modulated the increased risk of CHD with the differences of genetic background. Moreover, there was a decreased risk in Caucasians between the *ε*2 allele variant and CHD risk but no evidence in Mongolians; further studies should be conducted to verify it.

Our study has several limitations. First, as with all meta-analyses, although we did Egger's test and calculated the fail-safe number to evaluate the publication bias, it might have occurred because our analyses were all based on published studies. For the* ApoEε*4 compared with *ε*3 allele, the Egger test showed existence of publication bias, but from the results of the trim and fill method and the fail-safe number, the publication bias and the possibility of false positive were relatively small. Second, the control group of some studies was not in conformity with Hardy-Weinberg equilibrium. But, in sensitivity analysis, the pooled estimates were similar after we excluded studies deviating from Hardy-Weinberg equilibrium among controls; therefore these studies were included in the final analysis. Gene-environment interactions may have contributed to the CHD.* Apo E* gene is a candidate gene and a common one to study gene-environment interactions. However, because of lack of the original data of the meta-analysis, further evaluation of potential gene-gene and gene-environment interactions was limited.

In conclusion, it was showed in this meta-analysis that* ApoEε*4 allele polymorphism may contribute to the individual susceptibility of CHD. Further rigorous design, large sample of case-control, or prospective study are required to continue in-depth evaluation on gene-gene and gene-environment interactions on* Apo E* polymorphisms and CHD risk.

## Figures and Tables

**Table 1:** Distribution of the CHD cases and controls selection in the studies of APOE gene polymorphisms and CHD risk.

First author (year)	Ethnic	Case distribution	Percentage (%)	Control distribution	Percentage (%)	HW (*P*value)	NOS score
(1) Wang (2004)	Mongoloid (China)	146 cases of coronary heart disease confirmed by coronary angiography: 90 male cases and 56 females, mean ages 64 ± 11 years	30.0	340 control people had noncardiovascular disease and removed the body lipid metabolism disorder, cancer, and cerebrovascular disease population; 184 men and 156 women, mean age 63 ± 12 years	70.0	0.410	6
(2) Bai (2001)	Mongoloid (China)	All 65 patients with coronary heart disease patients were men, aged 47.16 ± 8.13 years on average	58.0	47 controls were noncardiovascular disease; mean age 46 ± 6 years	42.0	0.939	7
(3) Wang (2004)	Mongoloid (China)	110 cases were male and 76 females, aged 41 to 88 years, mean ages 65.0 ± 10.6 years	34.7	350 controls (185 males, 165 females), aged 40 to 87 years, mean 63.56 ± 8.32 years, were in the same hospital during the same period by the past medical history, physical examination, and ECG and other methods of noncardiac diagnosis of vascular disease cases	65.3	0.432	7
(4) Peng (2001)	Mongoloid (China)	213 cases of coronary heart disease and 52 cases of early-onset CHD and 161 cases of late CHD; early-onset group included 30 males and 22 females, mean age 47.4 ± 3.9 years; the late CHD group included 97 male cases and 64 female cases, mean age 69.8 ± 7.9 years	54.2	180 healthy persons acted as controls: 94 males and 86 females, mean age 53.1 ± 5.7 years	45.8	0.436	8
(5) Yang (2001)	Mongoloid (China)	204 cases of myocardial infarction and angina pectoris (coronary angiography showed stenosis greater than or equal to 70%) patients, 55 cases of early-onset group, the average age of 45.14 ± 8.18 years, 136 cases of late-onset group, the average age of 70.1 ± 6.4 years	60	136 control patients from the local companies and confirmed noncoronary heart disease; the average age of 52.15 ± 12.18 years	40	0.738	7
(6) Ouyang (2005)	Mongoloid (China)	A total of 200 cases of coronary heart disease; 105 cases were male and 95 cases were female; average age (63.6 ± 5.8) years	66.7	100 healthy people as control group: male 55 and female 45; average age 62.1 ± 7.8 years; no coronary heart disease symptoms; ECG and echocardiography tests were normal	33.3	0.399	7
(7) Heide (2009)	Caucasian (Germany)	121 cases of coronary heart disease: 100 cases of men, average age 33.7 ± 5.8 years; 21 females, mean age 39.1 ± 5.4 years	32.6	250 blood donors as controls, mean age 31.8 ± 6.7 years	67.4	0.529	8
(8) Kolovou (2009)	Caucasian (Greek)	359 cases were male patients with coronary heart disease; coronary angiography showed occlusion greater than or equal to 50%	59	248 health people were recruited as control, with no cardiovascular disease and no family history of cardiovascular disease	41	0.579	7
(9) Bahri (2008)	Caucasian (Tunisian)	80 cases (diagnosed after coronary angiography and ECG): 64 males and 16 females, mean age 57.42 ± 8.37 years; 56 were smoking, 51 had diabetes, 35 had hypertension, and 22 had high blood lipid	44.4	100 controls without coronary heart disease and other cardiovascular diseases, and cases from the same region in which 24 men and 76 women were randomly selected	55.6	0.815	6
(10) Akanji (2007)	Caucasian (Kuwaiti)	50 cases had evidence of acute myocardial infarction, the average age of 54 years	43.5	65 controls were randomly selected blood donors; the average age was 39 years without CHD family history; physical examination and blood examination ruled out other systemic diseases	56.5	0.441	7
(11) Aydogan (2009)	Caucasian (Turkey)	41 cases: 19 women and 22 men have undergone coronary angiography; patients with atherosclerosis risk factors such as diabetes, high blood pressure, and smoking	55.4	33 controls: 12 males and 11 females, no family history of coronary heart disease and coronary heart disease symptoms	44.6	0.754	7
(12) Ranjith (2004)	Caucasian (India)	195 cases were patients with acute myocardial infarction	39.4	300 controls from the same community, without cardiovascular disease and no associated risk factors	60.6	<0.001	7
(13) Peng (2003)	Mongoloid (China)	150 cases were diagnosed after coronary angiography or myocardial infarction	48.9	157 control patients from the hospital's medical examination, age and sex matched with cases	51.1	0.424	8
(14) Singh (2008)	Caucasian (India)	193 cases are diagnosed after coronary angiography; the average age of 54.94 ± 11.43 years	56.3	150 patients: 105 males and 45 females, mean age 53.42 ± 12.47 years	43.7	0.327	6
(15) Keavney (2003)	Caucasian	4484 cases of patients, mean age 50.5 ± 7.3 years, of which 1706 are smokers	43.8	5757 cases were of an average age of 45.7 ± 9.7 years, including 1151 smokers	56.2	0.463	8
(16) Freitas (2002)	Caucasian	640 cases were diagnosed by coronary angiography and (or) a history of myocardial infarction, mean age 44 ± 4 years, male 561, female 79, and 300 smokers	50.6	624 healthy people were randomly selected from the same community; no history of cardiovascular disease, mean age 40 ± 6 years, 322 men, 302 women, 183 smokers	49.4	0.767	8
(17) Kawakami (2001)	Mongoloid (Japan)	225 cases, of which 76 were vasospasm angina patients and 149 were ischemic heart disease, male 171, female 54	51.4	213 controls, average age 58.4 years, 152 male, 61 female; they were randomly selected from the hospital when they had an annual physical examination; no history of cardiovascular disease	48.6	0.510	7
(18) Attila (2001)	Caucasian (Turkey)	107 patients have undergone coronary angiography: 73 men and 34 women; the average age of 55.1 ± 10.2 years, 53 patients with hypertension, 22 with diabetes, 52 smokers	53.8	92 controls, after coronary angiography for the exclusion of noncoronary heart disease, of whom 51 were men and 41 women, average age 51.6 ± 9.5 years, 33 patients with hypertension, 6 with diabetes mellitus, 29 smokers	46.2	<0.001	6
(19) Kharrazi (2006)	Caucasian (Iran)	115 cases of 34 men and 81 women, mean age 54.4 ± 8.9 years	46	135 controls: 50 females and 85 males, mean age 55 ± 12.3 years	54	0.834	7
(20) Gamboa (2008)	Caucasian (Mexico)	156 patients, 124 males, 32 females, mean age 56.2 ± 9.8 years	43.8	200 controls: 150 males and 50 females, mean age 55.7 ± 4.16 years	56.2	<0.001	8
(21) Al-Yahyaee (2007)	Caucasian	67 cases who were diagnosed as rheumatoid arthritis patients with coronary heart disease	27.5	177 controls were cases of noncoronary heart disease patients with rheumatoid arthritis	72.5	0.903	6
(22) Al-Bustan (2009)	Caucasian (Kuwaiti)	143 patients: 91 were males and 52 females; mean age was 60.88 ± 12.1 years	54.0	122 controls: 65 males and 57 females, mean age 57.18 ± 13.0 years	46.0	<0.001	6
(23) Vaisi-Raygani (2010)	Caucasian (Iran)	162 patients were diagnosed by angiographic documented CAD and without diabetes 89 males and 73 females, mean age 56.3 ± 8.5 years	47.5	179 unrelated control subjects: 88 males and 91 females, mean age 55.7 ± 12.9 years	52.5	0.035	7
(24) Corella (2011)	Caucasian (Spain)	534 cases were participants who developed an incident CHD event during follow-up	32.2	1123 controls were healthy matched subjects	67.8	0.169	8
(25) Fallah (2011)	Caucasian (Iran)	190 patients: 140 males and 50 females; age range: 49–70 years	48.7	200 controls: 100 males and 100 females, age range: 36–62 years	51.3	0.059	6
(26) Gustavsson (2012)	Caucasian (Sweden)	1735 CHD cases included the INTERGENE study with upper age limit of 75 years and the SHEEP study with age of 45–70 years	27.2	4654 controls: the INTERGENE study: 3614 people aged 25–74 years; the SHEEP study: 1561 control subjects free from earlier MI diagnosis and matched for age and sex	72.8	0.765	7
(27) Heidari (2013)	Caucasian (Iran)	66 patients with significant lesions, male gender 35, mean age ± SD 52.5 ± 7.9	52.4	60 controls with normal coronary artery, male gender 32, mean age ± SD 51.2 ± 7.1	47.6	<0.001	6
(28) Marrzoq (2011)	Caucasian (Czech)	69 subjects (24 female and 45 male) with coronary artery disease	50.4	68 controls: 35 female and 33 male	49.6	0.708	6
(29) Afroze (2015)	Caucasian (India)	200 CAD patients (130 female and 70 male) were recruited from a cohort of patients undergoing clinically indicated coronary angiography	30.8	450 control subjects (260 female and 190 male)	69.2	<0.001	8
(30) Sapkota (2015)	Caucasian (USA)	723 CAD patients were diagnosed by nitrate medication, electrocardiographic evidence of angina pain, coronary angiographic evidence of severe (greater than 50%) stenosis, or echocardiographic evidence of myocardial infarction	37.4	1212 controls were healthy subjects without T2D or CAD	62.6	0.08	7

**Table 2:** Distribution of *Apo E* alleles in the case and control groups included in the meta-analysis.

First author (year)	Case/control (*N*/*N*)	Distribution of apolipoprotein E alleles					
		*ε*2		*ε*3		*ε*4	
		Case	Control	Case	Control	Case	Control
		*N* (%)	*N* (%)	*N* (%)	*N* (%)	*N* (%)	*N* (%)
(1) Wang (2004)	292/680	21 (7.2)	48 (7.1)	232 (79.4)	583 (85.7)	39 (13.4)	49 (7.2)
(2) Bai (2001)	100/94	4 (4.0)	5 (5.3)	90 (90.0)	86 (91.5)	6 (6.0)	3 (3.2)
(3) Wang (2004)	372/700	27 (7.3)	50 (7.1)	291 (78.2)	601 (85.9)	54 (14.5)	49 (7.0)
(4) Peng (2001)	426/360	34 (8.0)	30 (8.3)	328 (77.0)	303 (84.2)	64 (15.0)	27 (7.5)
(5) Yang (2001)	408/272	20 (4.9)	15 (5.5)	348 (85.3)	239 (87.9)	40 (9.8)	18 (6.6)
(6) Ouyang (2005)	400/200	31 (7.7)	18 (9.0)	291 (72.8)	167 (81.5)	78 (19.5)	19 (9.5)
(7) Heide (2009)	242/500	11 (4.5)	36 (8.3)	202 (83.5)	406 (81.2)	29 (12.0)	58 (11.5)
(8) Kolovou (2009)	718/496	37 (5.2)	39 (7.9)	612 (85.2)	414 (83.4)	69 (9.6)	43 (8.7)
(9) Bahri (2008)	160/200	6 (3.7)	9 (4.5)	140 (87.6)	174 (87.0)	14 (8.7)	17 (8.5)
(10) Akanji (2007)	100/130	6 (6.0)	9 (6.9)	87 (87.0)	108 (83.1)	7 (7.0)	13 (10.0)
(11) Aydogan (2009)	82/46	4 (4.9)	4 (17.4)	69 (84.1)	37 (80.4)	9 (11.0)	5 (11.6)
(12) Ranjith (2004)	390/600	10 (2.6)	27 (4.5)	330 (84.6)	517 (86.2)	50 (12.8)	56 (9.3)
(13) Peng (2003)	300/314	24 (8.0)	16 (5.0)	237 (79.0)	275 (87.6)	39 (13.0)	23 (7.4)
(14) Singh (2008)	386/300	21 (5.4)	23 (7.7)	307 (79.5)	257 (85.6)	58 (15.1)	20 (6.7)
(15) Keavney (2003)	8968/11514	609 (6.8)	911 (7.9)	6778 (75.6)	8830 (76.7)	1581 (17.6)	1773 (15.4)
(16) Freitas (2002)	1280/1248	61 (4.8)	90 (7.2)	1002 (78.2)	958 (76.8)	217 (17.0)	200 (16.0)
(17) Kawakami (2001)	450/426	13 (2.9)	15 (3.5)	390 (86.7)	349 (81.9)	47 (10.4)	62 (14.6)
(18) Attila (2001)	214/184	26 (12.1)	15 (8.1)	166 (77.6)	163 (88.6)	22 (10.3)	6 (3.3)
(19) Kharrazi (2006)	230/270	18 (7.8)	11 (4.1)	169 (73.5)	250 (92.6)	43 (18.7)	9 (3.3)
(20) Gamboa (2008)	312/400	5 (1.6)	9 (2.2)	283 (90.7)	364 (91.0)	24 (7.7)	27 (6.8)
(21) Al-Yahyaee (2007)	134/354	1 (0.7)	6 (1.7)	127 (94.8)	335 (94.6)	6 (4.5)	13 (3.7)
(22) Al-Bustan (2009)	286/244	21 (7.4)	23 (9.4)	246 (86.0)	207 (84.9)	19 (6.6)	14 (5.7)
(23) Vaisi-Raygani (2010)	324/357	31 (9.6)	40 (11.3)	257 (79.3)	289 (80.8)	36 (11.1)	28 (7.9)
(24) Corella (2011)	1035/2246	42 (4.1)	123 (5.5)	891 (86.1)	1928 (85.8)	102 (9.9)	195 (8.7)
(25) Fallah (2011)	380/400	73 (19.2)	55 (13.8)	141 (37.1)	218 (54.5)	166 (43.7)	127 (31.8)
(26) Gustavsson (2012)	3470/9308	199 (5.7)	738 (7.9)	2672 (77.0)	7065 (75.9)	599 (17.3)	1505 (16.2)
(27) Heidari (2013)	132/122	16 (12.1)	14 (11.5)	110 (83.3)	108 (88.5)	6 (4.5)	0 (0)
(28) Marrzoq (2011)	138/136	5 (3.6)	7 (5.2)	114 (82.6)	119 (87.5)	19 (13.8)	10 (7.4)
(29) Afroze (2015)	400/900	26 (6.5)	66 (7.3)	291 (72.8)	732 (81.3)	83 (20.8)	102 (11.3)
(30) Sapkota (2015)	1446/2432	52 (3.6)	95 (3.9)	1285 (88.9)	2174 (89.4)	109 (7.5)	163 (6.7)

**Table 3:** Distribution of *Apo E *genotypes in case and control groups included in the meta-analysis.

First author (year)	Case/control (*N*/*N*)	Distribution of apolipoprotein E genotype											
		E2/2		E2/3		E2/4		E3/3		E3/4		E4/4	
		Case	Control	Case	Control	Case	Control	Case	Control	Case	Control	Case	Control
		*N* (%)	*N* (%)	*N* (%)	*N* (%)	*N* (%)	*N* (%)	*N* (%)	*N* (%)	*N* (%)	*N* (%)	*N* (%)	*N* (%)
(1) Wang (2004)	146/340	0 (0.0)	3 (0.9)	19 (13.0)	41 (12.1)	2 (1.3)	1 (0.3)	89 (61.0)	249 (73.2)	35 (24.0)	44 (12.9)	1 (0.7)	2 (0.6)
(2) Bai (2001)	50/47	0 (0.0)	0 (0.0)	4 (8.0)	5 (10.6)	0 (0.0)	0 (0.0)	40 (80.0)	39 (82.99)	6 (12.0)	3 (6.4)	0 (0.0)	0 (0.0)
(3) Wang (2004)	186/350	0 (0.0)	3 (0.9)	25 (13.4)	43 (12.3)	2 (1.1)	1 (0.3)	108 (58.1)	257 (73.4)	50 (26.9)	44 (12.6)	1 (0.5)	2 (0.6)
(4) Peng (2001)	213/180	0 (0.0)	0 (0.0)	29 (13.6)	27 (15.0)	5 (2.3)	3 (1.7)	123 (57.7)	126 (70.0)	53 (24.9)	24 (13.3)	3 (1.4)	0 (0.0)
(5) Yang (2001)	204/136	1 (0.5)	1 (0.7)	18 (8.8)	12 (9.0)	0 (0.0)	1 (0.7)	153 (25.0)	106 (77.9)	24 (11.8)	15 (11.0)	8 (3.9)	1 (0.7)
(6) Ouyang (2005)	200/100	1 (0.5)	0 (0.0)	28 (14.0)	17 (17.0)	1 (1.0)	1 (0.5)	98 (49.0)	66 (66.0)	67 (33.5)	14 (14.0)	5 (2.5)	2 (2.0)
(7) Heide (2009)	121/250	0 (0.0)	0 (0.0)	7 (5.8)	31 (12.4)	4 (3.3)	5 (2.0)	88 (72.7)	163 (65.2)	19 (15.7)	49 (19.6)	3 (2.5)	2 (0.8)
(8) Kolovou (2009)	359/248	2 (0.6)	0 (0.0)	26 (7.2)	36 (14.5)	7 (1.9)	3 (1.2)	268 (74.7)	171 (69.0)	50 (13.9)	36 (14.5)	6 (1.7)	2 (0.8)
(9) Bahri (2008)	80/100	0 (0.0)	0 (0.0)	6 (7.5)	8 (8.0)	0 (0.0)	1 (1.0)	61 (76.3)	78 (78.0)	13 (16.3)	13 (13.0)	0 (0.0)	0 (0.0)
(10) Akanji (2007)	50/65	0 (0.0)	0 (0.0)	6 (12.0)	9 (13.8)	0 (0.0)	0 (0.0)	37 (74.0)	43 (66.2)	7 (14.0)	13 (20.0)	0 (0.0)	0 (0.0)
(11) Aydogan (2009)	41/23	0 (0.0)	0 (0.0)	4 (9.8)	3 (13.0)	0 (0.0)	1 (4.3)	28 (68.3)	15 (65.2)	9 (22.0)	4 (17.4)	0 (0.0)	0 (0.0)
(12) Ranjith (2004)	195/300	0 (0.0)	3 (1.0)	7 (4.0)	18 (6.0)	3 (1.0)	3 (1.0)	139 (71.0)	228 (76.0)	45 (83.0)	43 (14.0)	1 (1.0)	5 (2.0)
(13) Peng (2003)	150/157	1 (0.7)	1 (0.6)	21 (14.0)	13 (8.3)	1 (0.7)	1 (0.6)	93 (62.0)	122 (77.7)	30 (20.0)	18 (11.5)	4 (2.7)	2 (1.7)
(14) Singh (2008)	193/150	1 (0.5)	1 (0.7)	15 (7.8)	19 (12.7)	4 (2.1)	2 (1.3)	123 (63.7)	112 (74.7)	46 (23.8)	14 (9.7)	4 (2.1)	2 (1.3)
(15) Keavney (2003)	4484/5757	34 (0.8)	44 (0.8)	440 (9.8)	686 (11.9)	101 (2.3)	137 (2.4)	2566 (57.2)	3384 (58.8)	1206 (26.9)	1376 (23.9)	137 (13.0)	130 (2.2)
(16) Freitas (2002)	640/624	5 (0.8)	4 (0.6)	37 (5.8)	67 (10.7)	14 (2.2)	15 (2.4)	396 (62.0)	372 (60.0)	173 (27.0)	147 (24.0)	15 (2.3)	19 (3.0)
(17) Kawakami (2001)	225/213	3 (1.2)	0 (0.0)	6 (2.7)	13 (6.1)	1 (0.4)	2 (0.9)	177 (78.7)	140 (65.7)	30 (1.3)	56 (26.3)	8 (3.6)	2 (0.9)
(18) Attila (2001)	107/92	9 (8.4)	4 (4.3)	7 (6.5)	7 (7.6)	0 (0.0)	0 (0.0)	71 (66.4)	75 (81.6)	18 (16.8)	5 (5.4)	2 (1.9)	1 (1.1)
(19) Kharrazi (2006)	115/135	0 (0.0)	0 (0.0)	18 (15.7)	11 (8.1)	0 (0.0)	0 (0.0)	54 (47.0)	115 (85.2)	43 (37.4)	9 (6.7)	0 (0.0)	0 (0.0)
(20) Gamboa (2008)	156/200	0 (0.0)	4 (2.0)	5 (3.2)	1 (0.5)	0 (0.0)	0 (0.0)	127 (81.4)	168 (84.0)	24 (15.4)	27 (13.5)	0 (0.0)	0 (0.0)
(21) Al-Yahyaee (2007)	67/177	0 (0.0)	0 (0.0)	1 (1.5)	6 (3.4)	0 (0.0)	0 (0.0)	60 (89.6)	158 (89.3)	6 (9.0)	13 (7.3)	0 (0.0)	0 (0.0)
(22) Al-Bustan (2009)	143/122	8 (5.6)	9 (7.3)	3 (2.1)	2 (1.6)	2 (1.4)	3 (2.5)	114 (79.7)	98 (80.3)	15 (10.5)	9 (7.4)	1 (0.7)	1 (0.8)
(23) Vaisi-Raygani (2010)	162/179	0 (0)	6 (3.4)	31 (19.1)	26 (14.5)	0 (0)	2 (1.1)	99 (61.1)	119 (66.5)	28 (17.3)	24 (13.4)	4 (2.5)	2 (1.1)
(24) Corella (2011)	534/1123	1 (0.1)	1 (0)	33 (6.2)	105 (9.3)	7 (1.3)	16 (1.4)	400 (74.9)	828 (73.7)	91 (17.0)	167 (14.8)	2 (0.4)	6 (0.5)
(25) Fallah (2011)	190/200	13 (6.8)	7 (3.5)	10 (5.3)	22 (11.0)	37 (19.5)	19 (9.5)	35 (18.4)	59 (29.5)	61 (32.1)	78 (39.0)	34 (17.9)	15 (7.5)
(26) Gustavsson (2012)	1735/4654	11 (0.6)	32 (0.7)	133 (7.8)	547 (11.8)	44 (2.5)	127 (2.7)	1044 (60.2)	2689 (57.8)	451 (26.0)	1140 (24.5)	52 (3.0)	119 (2.6)
(27) Heidari (2013)	66/61	8 (12.1)	7 (11.5)	0 (0)	0 (0)	0 (0)	0 (0)	52 (78.8)	54 (88.5)	6 (9.1)	0 (0)	0 (0)	0 (0)
(28) Marrzoq (2011)	69/68	0 (0)	0 (0)	5 (7.2)	7 (10.3)	0 (0)	0 (0)	45 (65.2)	51 (75.0)	19 (27.5)	10 (14.7)	0 (0)	0 (0)
(29) Afroze (2015)	200/450	2 (1)	9 (2)	18 (9)	36	4 (2)	12 (2.7)	110 (55)	315 (7)	53 (26.5)	66 (14.7)	13 (6.5)	12 (2.7)
(30) Sapkota (2015)	723/1212	1 (0.1)	1 (0)	46 (6.4)	83 (6.8)	4 (0.5)	10 (0.8)	572 (79.1)	977 (80.6)	95 (13.1)	137 (11.3)	5 (0.7)	4 (0.3)
